# Deciphering the Active Phase Evolution of Co–W-Based Catalysts during Carbon Nanotube Growth by First-Principles Thermodynamics

**DOI:** 10.1021/acsnano.6c09726

**Published:** 2026-08-28

**Authors:** Ran Sun, Ting Cheng, Feng Ding, Lu Qiu

**Affiliations:** † Department of Materials Science and Engineering, 53025 City University of Hong Kong, Hong Kong 999077, China; ‡ Department of Chemistry, City University of Hong Kong, Hong Kong 999077, China; § Suzhou Laboratory, Suzhou 215123, China

**Keywords:** carbon nanotube, Co−W-based catalyst, phase evolution, density functional theory

## Abstract

Co–W-based catalysts represent one of the most promising systems for chirality-controlled carbon nanotube (CNT) growth. However, the identity of the active catalytic phase and its evolution under growth conditions remain under debate, leaving a critical gap in understanding how Co–W-based catalysts enable chirality-selective CNT synthesis. In this work, we investigated the thermodynamic stability and phase evolution of Co–W–C catalysts during CNT growth using first-principles calculations. By including vibrational contributions, we constructed phase diagrams of the Co–W–C system as a function of temperature, carbon chemical potential, and the W/Co ratio. The results show that Co_7_W_6_ is thermodynamically favored at low carbon chemical potential. Conversely, higher carbon chemical potential promotes the formation of carbide phases, such as Co_6_W_6_C, Co_2_W_4_C, and eventually Co_3_C, depending on temperature and catalyst composition. These diagrams consolidate previously reported active phases of Co–W-based catalysts (e.g., Co_7_W_6_, Co_6_W_6_C, Co–W–C η-carbide phases) into a single thermodynamic picture. Furthermore, the results also suggest that carbon supply may regulate the catalyst phase by changing the carbon chemical potential within the particle. This work clarifies the dynamic nature of Co–W–C catalyst evolution and establishes a thermodynamic framework for understanding phase behavior in Co–W-based catalysts for chirality-controlled CNT synthesis.

## Graphical abstract

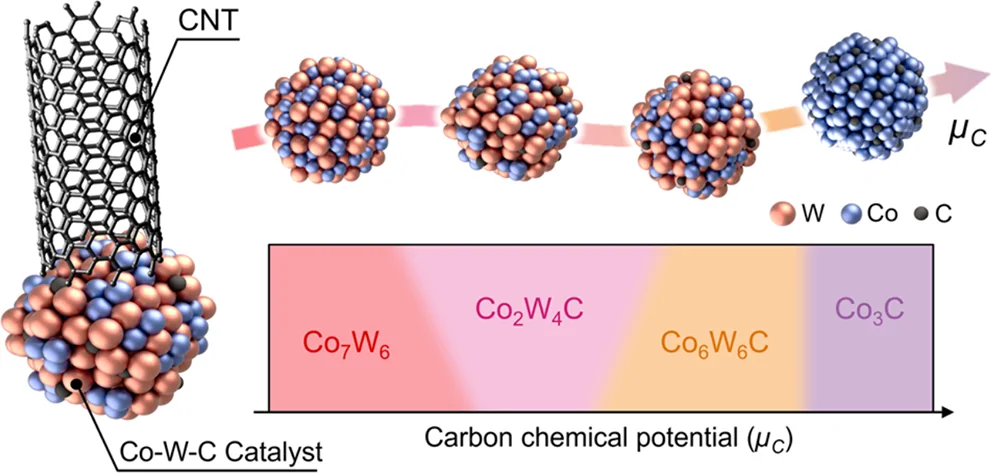

## Introduction

Since the discovery of carbon nanotubes (CNTs), many synthetic approaches have been developed because of their unique material properties^1–8^ and wide-ranging applications.^9–20^ The structure of a CNT is defined by its chiral indices (*n*, *m*), which determine the tube diameter and the wrapping angle. These structural variations control the intrinsic electronic properties of CNTs, including whether they are metallic or semiconducting. Therefore, the synthesis of structurally pure CNTs remains a significant challenge.

Chirality-selective CNT synthesis has remained a central goal for more than two decades. Currently, there are two main routes: postgrowth separation and direct chirality-selective growth. Postgrowth separation isolates specific CNT species from as-grown mixtures using solvents, surfactants, polymers, or DNA-based recognition systems.^21–27^ Although these methods yield impressive purities, their reliance on complex, multistep processing, high cost, and chemical additives limits their scalability and practical application.^7,28,29^

Direct chirality-selective growth aims to control CNT structure during synthesis, thereby avoiding postsynthetic separation. Several strategies have been developed, including vapor-phase epitaxy cloning^30^ and bottom-up growth from molecular templates.^31^ Among them, catalyst-mediated growth based on conventional chemical vapor deposition remains one of the most widely used routes because of its relatively simple operation, excellent scalability, tunable growth conditions, and broad compatibility with diverse catalyst systems.^32–34^ Accordingly, many catalysts have been explored for chirality-selective CNT growth, with a particular emphasis on bimetallic catalysts such as CoMo,^35^ NiFe,^36^ and FeCu.^37^

It is worth noting that Co_7_W_6_, a bimetallic solid-state catalyst, has emerged as a promising candidate for chirality-controlled CNT growth. Yang et al. reported that Co_7_W_6_ catalysts could yield a single dominant chirality with an unprecedented abundance of up to 92%.^38^ This highly efficient catalyst soon attracted considerable attention. In subsequent studies, different Co–W–C related phases were observed or proposed under CNT growth conditions. Yang et al. primarily attributed the chirality selectivity to the Co_7_W_6_ phase,^38–41^ whereas An et al. suggested that Co_6_W_6_C and Co might serve as active phases during synthesis.^42,43^ More recently, using in situ transmission electron microscopy, Wang et al. proposed the involvement of M_6_C-type η-carbide phases, such as Co_2_W_4_C and Co_3_W_3_C.^44^ These findings indicate that the Co–W–C system may undergo complex structural and phase evolution during CNT growth. Nevertheless, the mechanisms behind how these phases emerge, transform, and relate to each other under growth conditions remain poorly understood. This limits our mechanistic understanding of chirality-controlled CNT growth on Co–W-based catalysts and calls for a unified theoretical description of Co–W–C catalyst evolution during CNT growth.

In this study, we investigated the thermodynamic stability and phase evolution of Co–W–C catalysts during CNT growth using density functional theory (DFT) calculations. We considered a comprehensive set of experimentally reported and potentially relevant phases in the Co–W–C system, including graphite, Co, W, WC, W_2_C, Co_3_C, Co_7_W_6_, Co_6_W_6_C, Co_2_W_4_C, Co_3_W_3_C, Co_4_W_2_C, and Co_3_W_9_C_4_. By incorporating vibrational contributions, we evaluated their formation free energies and constructed Co–W–C phase diagrams. The resulting diagrams show how the catalyst evolves from Co_7_W_6_ to Co_6_W_6_C, Co_2_W_4_C, and Co_3_C with changes in temperature, carbon chemical potential, and catalyst composition. This provides a unified thermodynamic framework for rationalizing the experimentally observed Co–W alloy and carbide phases. These results further indicate that carbon incorporation into the catalyst drives the transition from Co–W alloy phases to Co–W–C carbide phases. Based on this understanding, we discuss the possibility of tuning the active catalyst phase by regulating carbon supply during growth, thereby offering a theoretical basis for understanding chirality-selective CNT synthesis on Co–W-based catalysts.

## Results and Discussion

Figure 1 presents the optimized lattice structures and calculated free energies of representative Co–W–C phases reported in previous studies. These phases include graphite, W, Co, WC, W_2_C, Co_3_C, Co_7_W_6_,^38^ and several ternary Co–W–C phases, such as M_12_C-type Co_6_W_6_C,^42^ M_6_C-type Co_2_W_4_C, Co_3_W_3_C, Co_4_W_2_C,^44^ and Co_3_W_9_C_4_. The optimized lattice structures of all considered phases are presented in the Supporting Information 1 (Figures S1–S12, Tables S1–S2), with Table S2 comparing our calculated lattice parameters to experimental data and showing excellent agreement. To incorporate the temperature effect, the free energies shown in Figure 1m were obtained by combining electronic internal energy with vibrational entropy from phonon calculations (see the Methods section). The calculated free energy curves allow an initial comparison of thermodynamic stability among the considered phases. Co_2_W_4_C has the lowest free energy, followed by Co_3_W_9_C_4_, Co_6_W_6_C, and Co_7_W_6_, whereas Co_3_W_3_C and Co_4_W_2_C are relatively less favorable. Although all free energies decrease with increasing temperature, differences in slope indicate that relative stabilities may change under actual growth temperatures.

**Figure 1:**
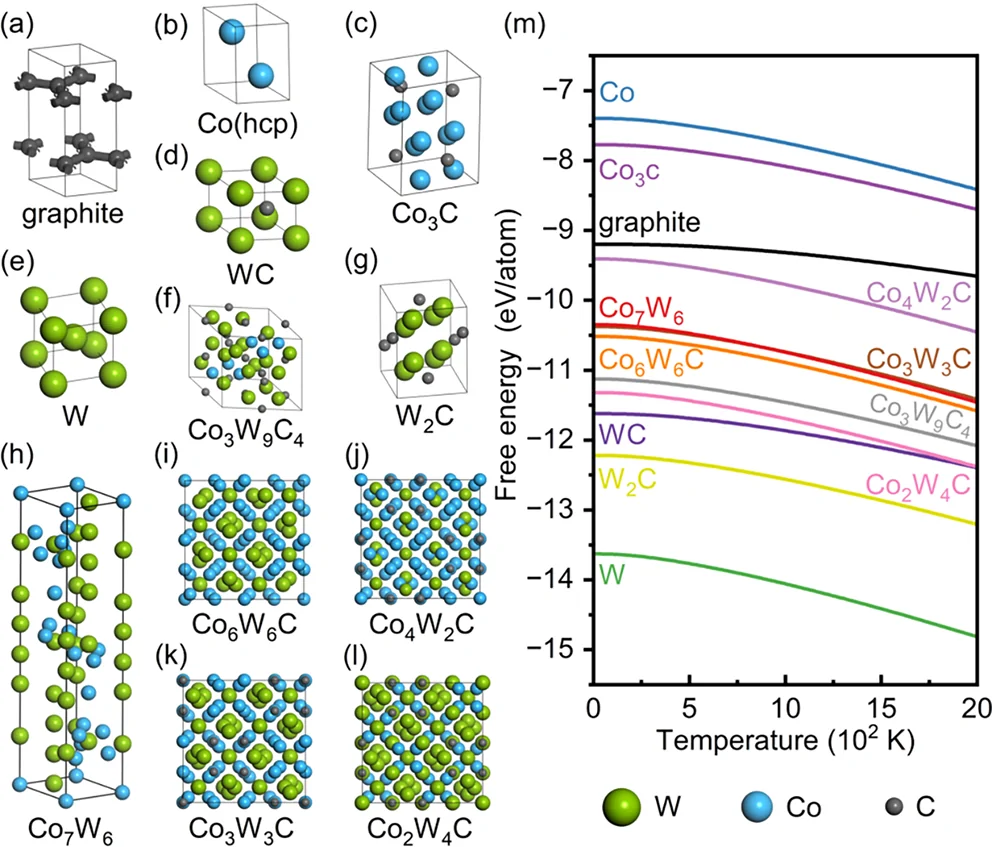
Optimized structures and free energies of possible phases in the Co–W–C system during CNT growth. (a–l) Optimized lattice structures of representative phases considered in this study, including graphite, W, Co, WC, W_2_C, Co_3_C, Co_7_W_6_, Co_6_W_6_C, Co_2_W_4_C, Co_3_W_3_C, Co_4_W_2_C, and Co_3_W_9_C_4_. (m) Calculated absolute free energies as a function of temperature from DFT and phonon calculations. These absolute DFT energies are not directly comparable to cohesive energies, as they have different reference states. Green, blue, and dark gray spheres represent W, Co, and C atoms, respectively.

However, absolute free energies discussed above do not directly reflect the relative formation stability in the multicomponent Co–W–C system. To compare phases on an equal basis, we calculated formation free energies (Δ*F*_*f*_) using pure Co, pure W, and graphite as reference states. The formation free energy of a given Co_*x*_W_*y*_C_*z*_ phase was calculated as

1 $${\Delta F}_{f}\left({\mathrm{Co}}_{x}W_{y}C_{z}\right)=\frac{F\left({\mathrm{Co}}_{x}W_{y}C_{z}\right)-\mathrm{xF}\left(\mathrm{Co}\right)-\mathrm{yF}\left(W\right)-z\mu_{C}}{x+y+z}$$

Here, Δ*F*_*f*_(*Co*_*x*_ *W*_*y*_ *C*_*z*_) is the formation free energy of Co_*x*_W_*y*_C_*z*_ phase in eV per atom; *F*(*Co*_*x*_ *W*_*y*_ *C*_*z*_) is the absolute free energy from DFT and phonon calculations; *x*, *y*, and *z* are the numbers of Co, W, and C atoms in the crystal lattice, respectively; *F*(*Co*) and *F*(*W*) represent the absolute free energies of elemental Co and W, respectively, from Figure 1m; and μ_*C*_ is the carbon chemical potential, which we set to that of graphite (Δμ_C_ = 0) unless otherwise specified. This approach is standard in thermodynamic studies of materials.^45,46^

Figure 2a shows the formation free energies at Δμ_C_ = 0. It can be seen that different phases exhibit distinct temperature dependencies. Within the examined temperature range, the ternary carbides Co_2_W_4_C and Co_6_W_6_C have the lowest formation free energies, followed by Co_7_W_6_, Co_3_W_3_C and Co_4_W_2_C. In contrast, Co_3_W_9_C_4_ has a much higher formation free energy under most conditions (Figures S13–S20) and is therefore excluded from further analysis. Although Co_2_W_4_C and Co_6_W_6_C are the most energetically favorable at Δμ_C_ = 0, it is crucial to recognize that a minimal formation free energy does not guarantee that a phase will be the only one present during CNT growth. The actual catalyst phase depends on the thermodynamic competition among all possible phases under given conditions of catalyst composition and growth environment.

**Figure 2:**
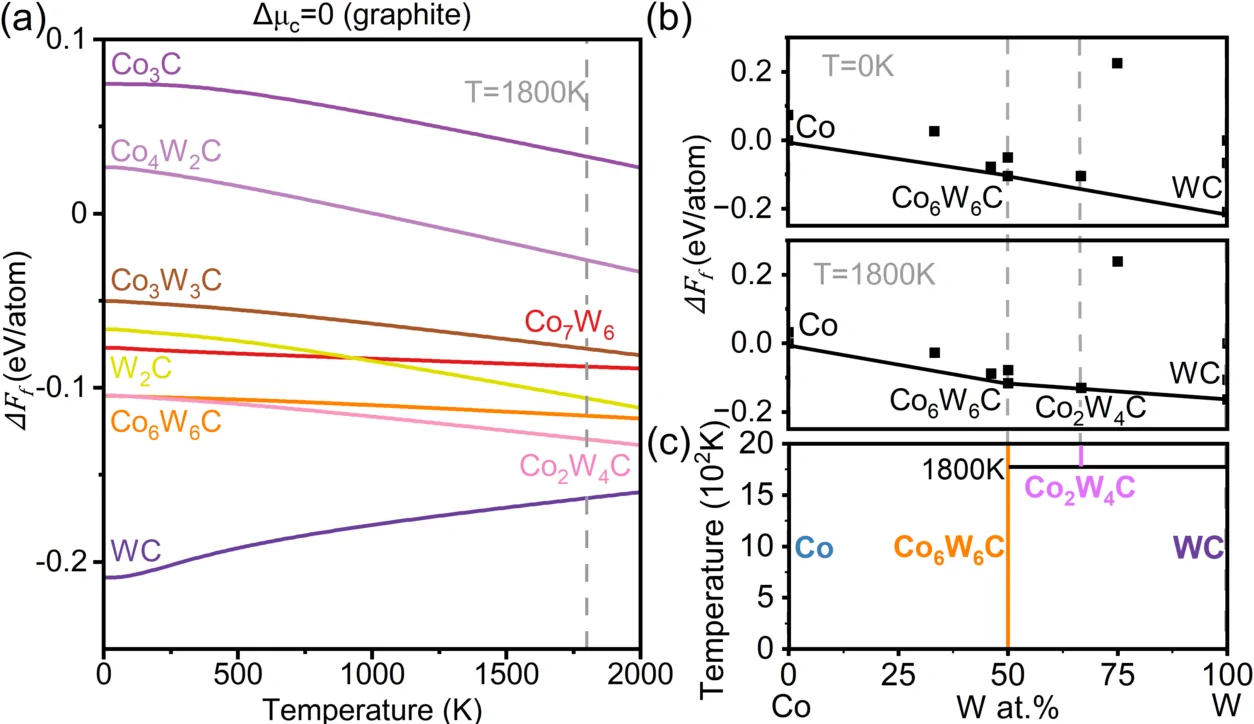
Formation free energies and phase diagram of the Co–W–C system at the graphite-referenced carbon chemical potential. (a) Formation free energies of phases in the Co–W–C system as a function of temperature, taking graphite as the carbon reference state (Δμ_C_ = 0). (b) Formation free energy plots of the considered phases as a function of W ratio among metal elements at 0 K and 1800 K. (c) Co–W–C phase diagram constructed at Δμ_C_ = 0 with graphite used as the carbon chemical potential.

To identify the potentially observed catalyst phases, we constructed convex-hull-type phase diagrams based on the formation free energies at all temperatures (see Supporting Information 2 for details). Figure 2b shows representative convex hull diagrams at 0 and 1800 K, plotted as a function of the W ratio. At 0 K, Co_6_W_6_C lies on the convex hull alongside Co and WC, indicating its thermodynamic stability. At 1800 K, both Co_6_W_6_C and Co_2_W_4_C appear on the hull, showing that Co_2_W_4_C becomes thermodynamically competitive at higher temperatures. Extending this analysis across the entire temperature range yields a comprehensive phase diagram with temperature and W ratio as variables (Figure 2c). When graphite is the reference state (Δμ_C_ = 0), Co_6_W_6_C is thermodynamically stable from 0 to 2000 K. It coexists with elemental Co in Co-rich regions and with WC in W-rich regions. Co_2_W_4_C appears only above approximately 1800 K and within W-rich regions (W ratio > 50%). These results confirm that temperature and W ratio together influence the stable phases in the Co–W–C system. While temperature shifts phase boundaries, the W ratio determines whether Co-rich, ternary carbides, or W-rich phases are favored.

It should be noted that, during actual CNT growth, the carbon chemical potential is not fixed at the graphite reference state. Instead, it changes dynamically due to carbon precursor decomposition, carbon diffusion into the catalyst, and CNT formation. Analysis restricted to Δμ_C_ = 0 alone cannot fully describe the complex phase transformation processes of Co–W-based catalysts under growth conditions. A comprehensive understanding requires evaluating phase stability across a broad range of carbon chemical potentials.

We therefore constructed Co–W–C phase diagrams as a function of carbon chemical potential (Figures S13–S20). During growth, continuous carbon supply increases the carbon chemical potential. As shown schematically in Figure 3a, the carbon chemical potential rises from low values in the precursor gas to higher values during CNT formation. Figure 3b-f shows the temperature-dependent formation free energies at different carbon chemical potentials. As expected, the formation free energies of all carbon-containing phases decrease as the carbon chemical potential increases. Under low carbon chemical potential, representing the initial gas environment, Co_7_W_6_ has the lowest formation free energy among all evaluated Co–W-containing phases. As the carbon chemical potential increases, carbide phases become more thermodynamically favorable. The formation free energy of WC decreases rapidly, while Co_6_W_6_C and Co_2_W_4_C become increasingly competitive relative to Co_7_W_6_.

**Figure 3:**
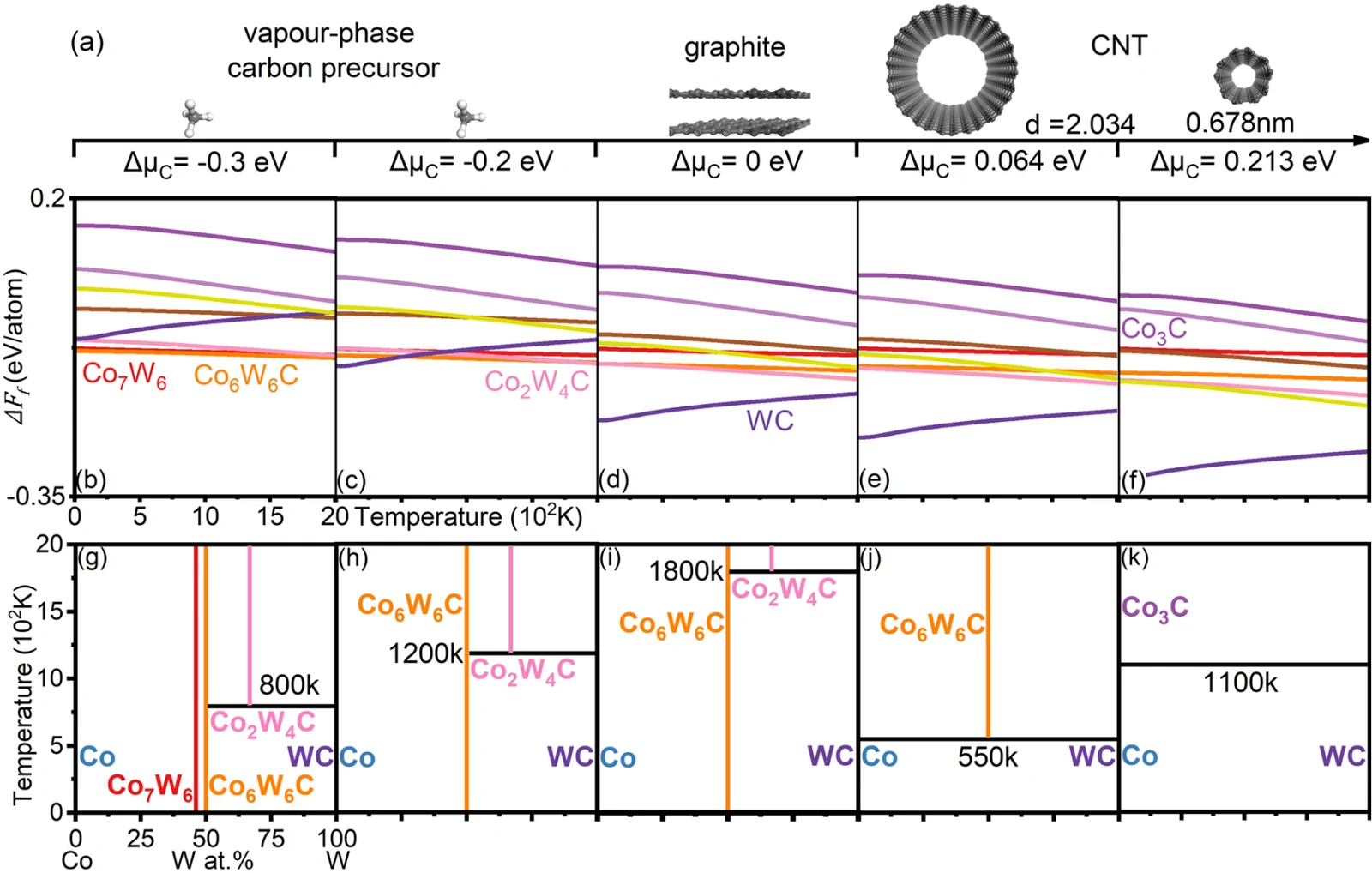
Formation free energies and phase diagrams of the Co–W–C system under different carbon chemical potentials. (a) Relative carbon chemical potential with graphite taken as the reference state (Δμ_C_ = 0), showing the variation from lower carbon chemical potential associated with carbon precursor gases to a higher value associated with CNTs. (b–f) Formation free energies of phases in the Co–W–C system as a function of temperature under different relative carbon chemical potentials. (g–k) Co–W–C phase diagrams constructed at the corresponding carbon chemical potentials.

Using the convex hull method from Figure 2, we constructed Co–W–C phase diagrams across a broad range of carbon chemical potentials (Figure 3g–k and Figures S13–S20). These diagrams show how the stable phases evolve with carbon chemical potential during CNT growth. In the initial vapor-phase environment with low carbon chemical potential, Co_7_W_6_ is predicted as the stable phase, while its stability region gradually disappears as the carbon chemical potential increases. Co_2_W_4_C and Co_6_W_6_C behave similarly. Specifically, Co_2_W_4_C is favored at high temperatures and W-rich compositions, but its phase field contracts as carbon chemical potential rises. Co_6_W_6_C is stable at lower carbon chemical potentials but destabilizes above Δμ_C_ = 0.213 eV. In contrast, WC and Co are robust over a wide range of temperatures and compositions. Only under high carbon chemical potential and high temperature is Co replaced by Co_3_C in the Co-rich region.

These phase diagrams illustrate the evolution of the Co–W–C system as carbon chemical potential increases, from Co_7_W_6_ to the ternary carbides Co_2_W_4_C and Co_6_W_6_C, and eventually to carbon-rich phases like Co_3_C. It should be noted that, although CNT formation corresponds to a relatively high carbon chemical potential, this does not mean that the catalyst always adopts the high-carbon phases. CNT growth is generally far from thermodynamic equilibrium, and kinetic factors, such as carbon diffusion, carbon feeding rate, and catalyst composition fluctuation, affect the actual phase. Therefore, these diagrams should be understood as a thermodynamic reference for interpreting the possible phase evolution of Co–W–C catalysts under CNT growth conditions.

In addition to temperature and carbon chemical potential, the catalyst composition, particularly the W/Co ratio, also influences phase behavior. Changes in this ratio may stabilize different alloy or carbide phases, directly affecting the active catalyst. Previous studies have shown that the W content in Co–W-based catalysts can change during CNT growth,^43^ highlighting the need to understand the composition-dependent phase evolution of the Co–W–C system. Therefore, we constructed phase diagrams spanning the entire range of W ratios (Figures S21–S25). Figure 4 shows three representative W/Co ratios. In these diagrams, the phase fields are plotted against carbon chemical potential (*x*-axis) and temperature (*y*-axis), with colored regions indicating stable phases.

**Figure 4:**
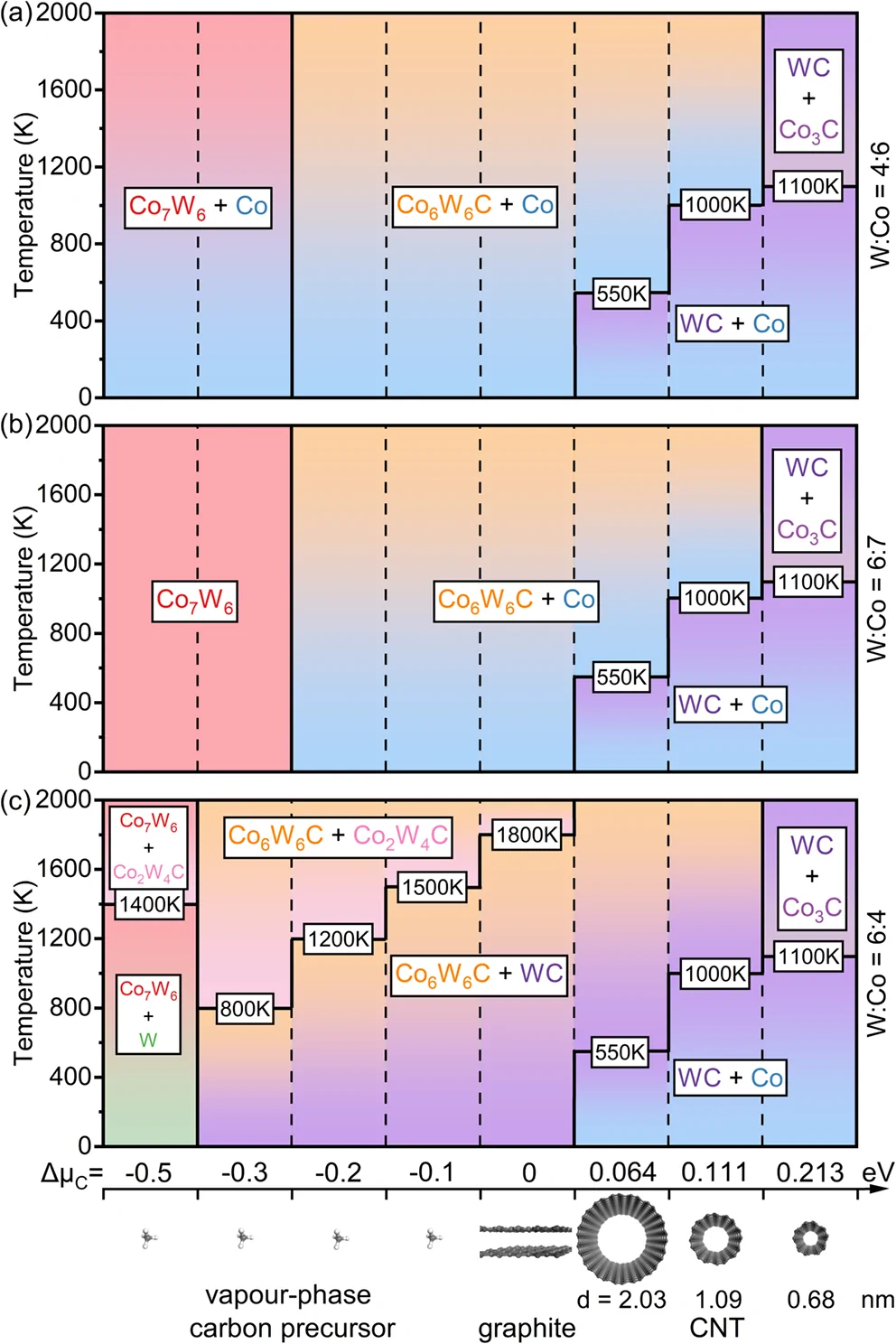
Phase diagrams of Co–W–C catalysts during CNT growth at different W/Co ratios. (a–c) Co–W–C phase diagrams constructed under different W/Co ratios among metal elements in the catalyst: (a) W:Co = 4:6, (b) W:Co = 6:7, and (c) W:Co = 6:4. The diagrams illustrate the phase evolution of Co–W–C catalysts as a function of temperature and carbon chemical potential under fixed catalyst compositions.

Figure 4a shows the phase diagram for a Co-rich catalyst with a W/Co ratio of 4:6. Co and Co_6_W_6_C are stable over broad regions, indicating that both Co-rich and ternary carbide phases are accessible during CNT growth. Co_7_W_6_ also coexists with Co over a specific carbon chemical potential range, suggesting that the alloy phase can form even when the initial W/Co ratio deviates from its nominal stoichiometry. This result agrees with An et al.,^43^ who reported that Co_6_W_6_C and Co phases are involved in CNT growth. Figure 4b presents the phase diagram for a W/Co ratio of 6:7, matching the stoichiometry of Co_7_W_6_. Here, Co_7_W_6_ occupies a distinct stability region, particularly at low carbon chemical potentials, consistent with Yang et al.^38^ In both cases, Co_7_W_6_ becomes less stable as the carbon chemical potential increases, while carbide phases become more favorable. This indicates that carbon incorporation drives the transformation from Co–W alloys to Co–W–C carbides.

For the W-rich catalyst with a W/Co ratio of 6:4 (Figure 4c), phase stability changes markedly. The stability region of Co_7_W_6_ becomes limited and often coexists with other phases. Instead, W-rich carbides become more favorable. Co_2_W_4_C appears within a certain carbon chemical potential window of approximately Δμ_C_ = −0.3 to 0 eV, especially at high temperatures. This suggests that W-enrichment promotes the formation of W-rich ternary carbides. The predicted appearance of Co_2_W_4_C aligns with Wang et al.,^44^ who observed M_6_C-type η-carbide phases in Co–W–C catalyst nanoparticles. Across all diagrams (Figure 4a-c), a consistent trend emerges: under high carbon chemical potential and high temperature, Co_3_C becomes thermodynamically favorable, agreeing with experimental reports of cobalt carbides during CNT growth.^47^

Overall, these composition-dependent phase diagrams show that the W/Co ratio strongly affects phase evolution. They reconcile different active phases reported in the literature and suggest that variations in catalyst composition can lead to different stable phases under growth conditions. Therefore, tuning the W/Co ratio provides an important route for regulating the active Co–W–C catalyst phase for chirality-selective CNT synthesis.

It should be noted that these calculations are based on bulk catalyst phases under a thermodynamic equilibrium. They do not explicitly account for the intrinsic finite-size effects of nanoscale catalyst particles or the nonequilibrium kinetics of CNT growth. Surface energy and particle shape can influence the relative stabilities of different phases. For instance, smaller nanoparticles, which have a higher surface-to-volume ratio, are known to be more prone to dissolve carbon atoms.^48^ This could potentially shift the phase boundaries and make phases stable at higher carbon chemical potentials (e.g., carbide phases) more favorable than our bulk predictions. While this work provides a robust thermodynamic framework, a quantitative investigation of these size-dependent effects remains an important direction for future research. Furthermore, the oxide supports commonly used in experiments represent a potential influencing factor. However, their impact on the active phase is likely to be limited. Previous studies suggested that while supports can affect the mobility of the catalyst nanoparticles, they do not appear to alter the intrinsic active phase of the catalyst.^47^ Nevertheless, the role of support interactions and their interplay with finite-size effects merit future investigations.

Based on these results, we propose that controlling carbon supply during CNT growth can regulate Co–W–C catalyst phases. Previous studies have shown that varying the carbon feeding rate modulates catalyst structures.^49^ Our phase diagrams suggest that the feeding rate controls the effective carbon chemical potential inside the catalyst through kinetic competition between CNT growth and inward carbon diffusion. As illustrated in Figure S26, a high carbon feeding rate may initiate growth before equilibrium is reached. Rapid carbon consumption for CNT growth at the surface outpaces inward bulk diffusion, resulting in a low internal carbon chemical potential and favoring Co_7_W_6_. An intermediate feeding rate allows partial carbon inward diffusion, increasing the internal carbon chemical potential and stabilizing ternary carbide phases such as Co_2_W_4_C and Co_6_W_6_C. At a low feeding rate, the system approaches quasi-equilibrium, allowing sufficient carbon diffusion and favoring carbon-rich phases like Co_3_C. Therefore, strategically modulating the carbon feeding rate thus offers an effective way to tune the active Co–W–C catalyst phase and thereby guide chirality-selective CNT growth.

## Conclusion

In this study, we used first-principles calculations to determine the thermodynamic stability and phase evolution of Co–W–C catalysts during CNT growth. By incorporating vibrational contributions to the free energy, we constructed phase diagrams to elucidate the catalyst phase evolution under CNT growth. By using carbon chemical potential and temperature as key variables, these phase diagrams reveal how the stable phases of the Co–W–C system evolve as carbon is introduced during CNT growth. In addition, phase diagrams constructed at different W/Co ratios demonstrate that catalyst composition strongly affects the accessible alloy and carbide phases.

The results thus identify temperature, carbon chemical potential, and W/Co ratio as three important factors governing the phase evolution of Co–W–C catalysts. Co_7_W_6_ is favored at relatively low carbon chemical potential, while increasing carbon chemical potential drives the sequential formation of ternary carbide phases such as Co_6_W_6_C, Co_2_W_4_C, and ultimately Co_3_C, depending on the specific temperature and catalyst composition. These findings provide a unified thermodynamic framework for understanding several active phases previously reported for Co–W-based catalysts.

Based on these phase diagrams, we further propose that the active Co–W–C catalyst phase can be tuned by regulating the carbon feeding rate, which controls carbon diffusion into the catalyst and thus the effective carbon chemical potential inside the catalyst. This strategy offers a possible route to control catalyst phase evolution and guide chirality-selective CNT growth. Overall, this work establishes a theoretical framework for understanding the rational design of Co–W-based catalysts toward controlled CNT synthesis.

## Method

In this study, DFT calculations were performed using Vienna Ab initio Simulation Package (VASP).^50,51^ The ion-electron interactions were described by the projector augmented-wave (PAW) method,^52^ while the exchange-correlation interactions were described using the Perdew–Burke–Ernzerhof (PBE) functional within the generalized gradient approximation (GGA).^53^ The Brillouin zone was sampled using γ-centered Monkhorst–Pack k-point meshes, with the *k*-point spacing limited to no more than 0.3 *Å* ^–1^. The convergence criteria for electronic energy and atomic force were set at 10^–4^eV and 10^–2^eV/Å, respectively. A plane-wave cutoff energy of 400 eV was used for structural optimization.

To obtain the temperature-dependent free energy for each phase, the electronic internal energy and vibrational contribution were calculated separately. The electronic internal energy was obtained directly from VASP calculations and represents the ground-state total energy of the optimized lattice at 0 K. The vibrational contribution was calculated using the finite displacement method. Specifically, supercell calculations with and without symmetry-adapted atomic displacements were first performed using VASP. The force constant matrix was then constructed using Phonopy^54,55^ to construct the dynamical matrix and extract the phonon frequencies. To ensure highly accurate force calculations for the phonon spectra, the convergence criteria for energy and atomic force tightened to 10^–8^eV and 10^–3^eV/Å, respectively, while the plane-wave cutoff energy was increased to 500 eV.

The free energy of each phase was calculated as follows

2 $$F\left({\mathrm{Co}}_{x}W_{y}C_{z}\right)=-k_{B}T\;\ln Z$$

3 $$F\left({\mathrm{Co}}_{x}W_{y}C_{z}\right)=\varphi +\frac{1}{2}\sum_{q\nu }\hbar \omega \left(q\nu \right)+k_{B}T\sum_{q\nu }\ln\left[1-\exp\left(\frac{-\hbar \omega \left(q\nu \right)}{k_{B}T}\right)\right]$$

where *F*(*Co*_*x*_ *W*_*y*_ *C*_*z*_) is the free energy of the Co_*x*_W_*y*_C_*z*_ phase; *k*_*B*_, *T*, and *Z* represent the Boltzmann constant, temperature, and partition function, respectively; and φ is the electronic internal energy calculated by VASP. The second term is the zero-point energy, corresponding to lattice vibrations at 0 K. The third term represents the finite-temperature vibrational contribution arising from thermally excited phonons. Here, ℏ, ω, *q*, and ν denote the reduced Planck constant, phonon angular frequency, phonon wave vector, and phonon branch index, respectively. The finite-temperature electronic contribution to the free energy is estimated to be negligible (less than 0.3% at 1000 K) for the phases and temperatures relevant to CNT growth (see Table S3). It is therefore omitted, and the free energy is calculated from the vibrational contributions alone.

## Supplementary Material



## Notes

Optimized lattices and lattice parameters, including a comparison of calculated and experimental lattice parameters for the key phases (Figures S1–S12 and Tables S1–S2); formation free energy calculation and construction of phase diagrams (Figures S13–S25); a possible strategy for regulating Co–W–C catalyst phases (Figure S26); and electronic contributions to the free energy for key phases (Table S3) (DOCX)
