# Supplementary material for: Deciphering the Active Phase Evolution of Co–W-Based Catalysts during Carbon Nanotube Growth by First-Principles Thermodynamics
Source: ACS Nano. 2026 Aug 28;20(36):24969–76. doi: 10.1021/acsnano.6c09726 (PMC13585179; doi:10.1021/acsnano.6c09726)
Supplement: Supplementary file 1 [file nn6c09726_si_001.docx]

Supplementary Information for

Deciphering the Active Phase Evolution of Co–W-based Catalysts during Carbon Nanotube Growth by First-Principles Thermodynamics

*Ran Sun^1^, Ting Cheng^2^, Feng Ding*^3^, Lu Qiu*^1^*

^1^Department of Materials Science and Engineering, City University of Hong Kong, Hong Kong, 999077, China

^2^Department of Chemistry, City University of Hong Kong, Hong Kong, 999077, China

^3^Suzhou Laboratory, Suzhou 215123, China

*Correspondence: *dingf@szlab.ac.cn*; *luqiu6@cityu.edu.hk*

Supplementary Information 1:

Optimized Lattices and Lattice Parameters

Figure S1. (a) Top view, (b) front view and (c) side view of optimized graphite lattice

Figure S2. (a) Top view, (b) front view and (c) side view of optimized Co lattice

Figure S3. (a) Top view, (b) front view and (c) side view of optimized Co_3_C lattice

Figure S4. (a) Top view, (b) front view and (c) side view of optimized WC lattice

Figure S5. (a) Top view, (b) front view and (c) side view of optimized W lattice

Figure S6. (a) Top view, (b) front view and (c) side view of optimized Co_3_W_9_C_4_ lattice

Figure S7. (a) Top view, (b) front view and (c) side view of optimized W_2_C lattice

Figure S8. (a) Top view, (b) front view and (c) side view of optimized Co_7_W_6_ lattice

Figure S9. (a) Top view, (b) front view and (c) side view of optimized Co_6_W_6_C lattice

Figure S10. (a) Top view, (b) front view and (c) side view of optimized Co_4_W_2_C lattice

Figure S11. (a) Top view, (b) front view and (c) side view of optimized Co_3_W_3_C lattice

Figure S12. (a) Top view, (b) front view and (c) side view of optimized Co_2_W_4_C lattice

**Table S1.** Optimized lattice parameters

| Structure | Lattice parameter | | | | | |
| --- | --- | --- | --- | --- | --- | --- |
|  | Lattice constants (Å) | | | Interaxial angles (°) | | |
|  | *a* | *b* | *c* | *α* | *β* | *γ* |
| graphite | 2.500 | 2.500 | 5.319 | 90 | 90 | 120 |
| Co | 2.491 | 2.491 | 4.032 | 90 | 90 | 120 |
| Co_3_C | 4.372 | 4.894 | 6.631 | 90 | 90 | 90 |
| WC | 2.900 | 2.900 | 2.832 | 90 | 90 | 120 |
| W | 3.151 | 3.151 | 3.151 | 90 | 90 | 90 |
| Co_3_W_9_C_4_ | 7.845 | 7.845 | 7.506 | 90 | 90 | 120 |
| W_2_C | 4.708 | 5.191 | 6.043 | 90 | 90 | 90 |
| Co_7_W_6_ | 4.700 | 4.700 | 25.303 | 90 | 90 | 120 |
| Co_6_W_6_C | 10.775 | 10.775 | 10.775 | 90 | 90 | 90 |
| Co_4_W_2_C | 10.999 | 10.632 | 10.632 | 90 | 90 | 90 |
| Co_3_W_3_C | 10.942 | 10.942 | 10.942 | 90 | 90 | 90 |
| Co_2_W_4_C | 11.197 | 11.197 | 11.197 | 90 | 90 | 90 |

**Table S2.** Comparison of calculated and experimental lattice parameters for the key phases Co_7_W_6_, Co_6_W_6_C, Co_2_W_4_C, and Co_3_W_3_C.

|  |  | Co_7_W_6_ | Co_6_W_6_C | Co_2_W_4_C | Co_3_W_3_C |
| --- | --- | --- | --- | --- | --- |
| Calculated lattice parameters (Å) | *a* | 4.700 | 10.775 | 11.197 | 10.942 |
|  | *b* | 4.700 | 10.775 | 11.197 | 10.942 |
|  | *c* | 25.303 | 10.775 | 11.197 | 10.942 |
| Reference lattice parameters (Å) | *a* | 4.72^1^ | 10.90^2^ | 11.21^2^ | 11.116^2^ |
|  | *b* | 4.72^1^ | 10.90^2^ | 11.21^2^ | 11.116^2^ |
|  | *c* | 25.50^1^ | 10.90^2^ | 11.21^2^ | 11.116^2^ |
| Error (%) | *a* | -0.42% | -1.15% | 0.12% | -1.57% |
|  | *b* | -0.42% | -1.15% | 0.12% | -1.57% |
|  | *c* | -0.77% | -1.15% | 0.12% | -1.57% |

Table S2 presents a comparison of our calculated lattice parameters for the key phases (Co_7_W_6_, Co_6_W_6_C, Co_2_W_4_C, and Co_3_W_3_C) with published experimental data.^1,2^ The comparison reveals only minor deviations, with all errors below 2%, well within a reasonable range and demonstrating good agreement between our theoretical results and experimental references.
Supplementary Information 2:

Formation Free Energy and Phase Diagrams

Figure S13 illustrates a method for plotting phase diagrams based on the formation free energies of Co-W-C phases. The calculated formation free energies under the graphite-referenced carbon chemical potential of vapour-phase carbon precursor ($\Delta\mu_{C}=-0.5$) are shown in Figure S13a. Based on these formation free energy data, we constructed convex-hull-type phase diagrams at each temperature. Figure S13b shows representative formation free energy plots at 0 K, 1400 K and 2000 K as a function of the W metallic fraction. At 0 K, Co_7_W_6_ lies on the convex hull of the curve and is therefore predicted to be a thermodynamically stable phase, similar to the endpoint phases Co and W. This trend continues up to a temperature of 1400 K. At 1400 K, both Co_7_W_6_ and Co_2_W_4_C occupy the convex hull of the curve, which suggests that Co_2_W4_C_ is competitive at such temperatures. Situations where Co, Co_7_W_6_, Co_2_W_4_C and W exhibit thermodynamic stability extend from 1400 K up to 2000 K. By extending this analysis over the entire temperature range, a phase diagram with temperature and W metallic fraction as variables was obtained as shown in Figure S13c. By plotting straight lines representing W/Co ratios of 4:6, 6:7 and 6:4 on the phase diagram in Figure S13c, we can visually demonstrate the active phases exhibited on the Co-W-C catalyst, as shown in Figure S13d. Using this method, we analyzed a series of carbon chemical potentials during CNT growth (Figures S13-S20), plotted phase diagrams with temperature and W metallic fraction as variables for each carbon chemical potential (Figures 3g-k), and summarized these into phase diagrams with temperature and carbon chemical potential as variables for different W/Co ratios (Figures 4a-c).

Figure S13. (a) Formation free energy of phases in the Co-W-C system as a function of temperature in a vapor-phase carbon precursor environment, taking graphite as a reference carbon chemical potential ($\Delta\mu_{C}=-0.5$). (b) Scatter charts of formation free energy of each phase at the lowest temperature (0 K), the phase transition temperature (1400 K) and the highest temperature (2000 K). (c) Co-W-C phase diagram at carbon chemical potential $\Delta\mu_{C}=-0.5$ taking graphite as a reference. (d) Active phases of Co-W-C catalysts with ratios of W:Co of 4:6, 6:7 and 6:4 at carbon chemical potential $\Delta\mu_{C}=-0.5$.

Figure S14. (a) Formation free energy of phases in the Co-W-C system as a function of temperature in a vapor-phase carbon precursor environment, taking graphite as a reference carbon chemical potential ($\Delta\mu_{C}=-0.3$). (b) Scatter charts of formation free energy of each phase at the lowest temperature (0 K), the phase transition temperature (800 K) and the highest temperature (2000 K). (c) Co-W-C phase diagram at carbon chemical potential $\Delta\mu_{C}=-0.3$ taking graphite as a reference. (d) Active phases of Co-W-C catalysts with ratios of W:Co of 4:6, 6:7 and 6:4 at carbon chemical potential $\Delta\mu_{C}=-0.3$.

Figure S15. (a) Formation free energy of phases in the Co-W-C system as a function of temperature in a vapor-phase carbon precursor environment, taking graphite as a reference carbon chemical potential ($\Delta\mu_{C}=-0.2$). (b) Scatter charts of formation free energy of each phase at the lowest temperature (0 K), the phase transition temperature (1200 K) and the highest temperature (2000 K). (c) Co-W-C phase diagram at carbon chemical potential $\Delta\mu_{C}=-0.2$ taking graphite as a reference. (d) Active phases of Co-W-C catalysts with ratios of W:Co of 4:6, 6:7 and 6:4 at carbon chemical potential $\Delta\mu_{C}=-0.2$.

Figure S16. (a) Formation free energy of phases in the Co-W-C system as a function of temperature in a vapor-phase carbon precursor environment, taking graphite as a reference carbon chemical potential ($\Delta\mu_{C}=-0.1$). (b) Scatter charts of formation free energy of each phase at the lowest temperature (0 K), the phase transition temperature (1500 K) and the highest temperature (2000 K). (c) Co-W-C phase diagram at carbon chemical potential $\Delta\mu_{C}=-0.1$ taking graphite as a reference. (d) Active phases of Co-W-C catalysts with ratios of W:Co of 4:6, 6:7 and 6:4 at carbon chemical potential $\Delta\mu_{C}=-0.1$.

Figure S17. (a) Formation free energy of phases in the Co-W-C system as a function of temperature taking graphite as a reference carbon chemical potential ($\Delta\mu_{C}=0$). (b) Scatter charts of formation free energy of each phase at the lowest temperature (0 K), the phase transition temperature (1800 K) and the highest temperature (2000 K). (c) Co-W-C phase diagram at carbon chemical potential $\Delta\mu_{C}=0$ taking graphite as a reference. (d) Active phases of Co-W-C catalysts with ratios of W:Co of 4:6, 6:7 and 6:4 at carbon chemical potential $\Delta\mu_{C}=0$.

Figure S18. (a) Formation free energy of phases in the Co-W-C system as a function of temperature in a CNT growth environment, taking graphite as a reference carbon chemical potential ($\Delta\mu_{C}=0.064$). (b) Scatter charts of formation free energy of each phase at the lowest temperature (0 K), the phase transition temperature (550 K) and the highest temperature (2000 K). (c) Co-W-C phase diagram at carbon chemical potential $\Delta\mu_{C}=0.064$ taking graphite as a reference. (d) Active phases of Co-W-C catalysts with ratios of W:Co of 4:6, 6:7 and 6:4 at carbon chemical potential $\Delta\mu_{C}=0.064$.

Figure S19. (a) Formation free energy of phases in the Co-W-C system as a function of temperature in a CNT growth environment, taking graphite as a reference carbon chemical potential ($\Delta\mu_{C}=0.111$). (b) Scatter charts of formation free energy of each phase at the lowest temperature (0 K), the phase transition temperature (1000 K) and the highest temperature (2000 K). (c) Co-W-C phase diagram at carbon chemical potential $\Delta\mu_{C}=0.111$ taking graphite as a reference. (d) Active phases of Co-W-C catalysts with ratios of W:Co of 4:6, 6:7 and 6:4 at carbon chemical potential $\Delta\mu_{C}=0.111$.

Figure S20. (a) Formation free energy of phases in the Co-W-C system as a function of temperature in a CNT growth environment, taking graphite as a reference carbon chemical potential ($\Delta\mu_{C}=0.213$). (b) Scatter charts of formation free energy of each phase at the lowest temperature (0 K), the phase transition temperature (1100 K) and the highest temperature (2000 K). (c) Co-W-C phase diagram at carbon chemical potential $\Delta\mu_{C}=0.213$ taking graphite as a reference. (d) Active phases of Co-W-C catalysts with ratios of W:Co of 4:6, 6:7 and 6:4 at carbon chemical potential $\Delta\mu_{C}=0.213$.

Figure S21. The Co-W-C phase diagram of a relatively Co-rich catalyst with a W ratio below 46.15%.

Figure S22. The Co-W-C phase diagram for a Co-W-based catalyst with a W:Co ratio of 6:7 (i.e. a W ratio of 46.15%).

Figure S23. The Co-W-C phase diagram for a Co-W-based catalyst with a W ratio between 46.15% and 50%.

Figure S24. The Co-W-C phase diagram for Co-W-based catalysts with a W ratio between 50% and 66.67%.

Figure S25. The Co-W-C phase diagram for a relatively W-rich catalyst with a W ratio above 66.67%.

Supplementary Information 3:

**A possible strategy for regulating Co–W–C catalyst phases**

Figure S26. Schematic illustration of the proposed strategy for regulating the active phase of Co–W–C catalysts during CNT growth. The carbon feeding rate influences carbon diffusion into the catalyst, thereby modifying the carbon chemical environment and controlling the active Co–W–C phase during CNT growth.

Supplementary Information 4:

| Phase | Co_2_W_4_C | Co_6_W_6_C | Co_7_W_6_ | Co_3_C | Co |
| --- | --- | --- | --- | --- | --- |
| Number of atoms in the unit cell | 112 | 104 | 39 | 16 | 2 |
| g(E_F_) (states/eV) | 107.56 | 199.01 | 27.770 | 9.5490 | 1.7280 |
| g(E_F_) (states/eV*atom) | 0.96036 | 1.9136 | 0.71205 | 0.59681 | 0.86400 |
| Electronic free energy at 500K (meV/atom) | -2.9325 | -5.8431 | -2.1743 | -1.8224 | -2.6382 |
| Electronic free energy at 1000K (meV/atom) | -11.730 | -23.372 | -8.6970 | -7.2895 | -10.553 |
| Electronic free energy at 1500K (meV/atom) | -26.392 | -52.588 | -19.568 | -16.401 | -23.744 |
| Electronic free energy at 2000K (meV/atom) | -46.920 | -93.489 | -34.788 | -29.158 | -42.212 |
| Free energy at 500K (meV/atom) | -11437 | -10632 | -10473 | -7861.9 | -7501.5 |
| Free energy at 1000K (meV/atom) | -11693 | -10890 | -10742 | -8082.6 | -7747.3 |
| Free energy at 1500K (meV/atom) | -12016 | -11214 | -11078 | -8369.4 | -8060.0 |
| Free energy at 2000K (meV/atom) | -12382 | -11582 | -11458 | -8699.7 | -8416.5 |
| Error at 500K | 0.026% | 0.055% | 0.021% | 0.023% | 0.035% |
| Error at 1000K | 0.10% | 0.21% | 0.081% | 0.090% | 0.14% |
| Error at 1500K | 0.22% | 0.47% | 0.18% | 0.20% | 0.29% |
| Error at 2000K | 0.38% | 0.80% | 0.30% | 0.33% | 0.50% |

**Table S3.** Electronic contributions to the free energy for key phases.

We first calculated the density of states at the Fermi level and used the Sommerfeld expansion,

$$\begin{aligned} F_{elec}\approx-\frac{\pi^{2}}{6}\left( k_{B}T \right)^{2}\cdot g\left( E_{F} \right)\#\left( S1 \right) \end{aligned}$$

to estimate the electronic free energy contribution ($F_{elec}$) for the most relevant phases (Co, Co_3_C, Co_7_W_6_, Co_6_W_6_C, Co_2_W_4_C). Table S3 shows the electron contribution to the free energy, the original free energy values and the relative errors across the entire temperature range. It can be seen that $F_{elec}$ is a very small fraction of the total free energy at all temperatures, with relative errors below 0.3% at 1000 K (the critical temperature range for CNT growth) and below 0.8% even at 2000 K.

**Reference**

(1) Yang, F.; Zhao, H.; Wang, X.; Liu, X.; Liu, Q.; Liu, X.; Jin, C.; Wang, R.; Li, Y. Atomic scale stability of tungsten–cobalt intermetallic nanocrystals in reactive environment at high temperature. *Journal of the American Chemical Society* **2019**, *141* (14), 5871–5879.
(2) Wang, Y.; Qiu, L.; Zhang, L.; Tang, D.-M.; Ma, R.; Ren, C.-L.; Ding, F.; Liu, C.; Cheng, H.-M. Growth mechanism of carbon nanotubes from Co-W-C alloy catalyst revealed by atmospheric environmental transmission electron microscopy. *Science Advances* **2022**, *8* (49), eabo5686.
